# Liquid temperature prediction in bubbly flow using ant colony optimization algorithm in the fuzzy inference system as a trainer

**DOI:** 10.1038/s41598-020-78751-y

**Published:** 2020-12-14

**Authors:** Meisam Babanezhad, Iman Behroyan, Ali Taghvaie Nakhjiri, Azam Marjani, Amir Heydarinasab, Saeed Shirazian

**Affiliations:** 1 Institute of Research and Development, Duy Tan University, Da Nang, 550000 Vietnam; 2 Faculty of Electrical-Electronic Engineering, Duy Tan University, Da Nang, 550000 Vietnam; 3 Mechanical and Energy Engineering Department, Shahid Beheshti University, Tehran, Iran; 4 Department of Petroleum and Chemical Engineering, Science and Research Branch, Islamic Azad University, Tehran, Iran; 5 Department for Management of Science and Technology Development, Ton Duc Thang University, Ho Chi Minh City, Vietnam; 6 Faculty of Applied Sciences, Ton Duc Thang University, Ho Chi Minh City, Vietnam; 7 Laboratory of Computational Modeling of Drugs, South Ural State University, 76 Lenin Prospekt, 454080 Chelyabinsk, Russia

**Keywords:** Chemical engineering, Mechanical engineering, Computational science, Computer science, Software

## Abstract

In the current research paper a novel hybrid model combining first-principle and artificial intelligence (AI) was developed for simulation of a chemical reactor. We study a 2-dimensional reactor with heating sources inside it by using computational fluid dynamics (CFD). The type of considered reactor is bubble column reactor (BCR) in which a two-phase system is created. Results from CFD were analyzed in two different stages. The first stage, which is the learning stage, takes advantage of the swarm intelligence of the ant colony. The second stage results from the first stage, and in this stage, the predictions are according to the previous stage. This stage is related to the fuzzy logic system, and the ant colony optimization learning framework is build-up this part of the model. Ants movements or swarm intelligence of ants lead to the optimization of physical, chemical, or any kind of processes in nature. From point to point optimization, we can access a kind of group optimization, meaning that a group of data is studied and optimized. In the current study, the swarm intelligence of ants was used to learn the data from CFD in different parts of the BCR. The learning was also used to map the input and output data and find out the complex connection between the parameters. The results from mapping the input and output data show the full learning framework. By using the AI framework, the learning process was transferred into the fuzzy logic process through membership function specifications; therefore, the fuzzy logic system could predict a group of data. The results from the swarm intelligence of ants and fuzzy logic suitably adapt to CFD results. Also, the ant colony optimization fuzzy inference system (ACOFIS) model is employed to predict the temperature distribution in the reactor based on the CFD results. The results indicated that instead of solving Navier–Stokes equations and complex solving procedures, the swarm intelligence could be used to predict a process. For better comparisons and assessment of the ACOFIS model, this model is compared with the genetic algorithm fuzzy inference system (GAFIS) and Particle swarm optimization fuzzy inference system (PSOFIS) method with regards to model accuracy, pattern recognition, and prediction capability. All models are at a similar level of accuracy and prediction ability, and the prediction time for all models is less than one second. The results show that the model’s accuracy with low computational learning time can be achieved with the high number of CIR (0.5) when the number of inputs ≥ 4. However, this finding is vice versa, when the number of inputs < 4. In this case, the CIR number should be 0.2 to achieve the best accuracy of the model. This finding could also highlight the importance of sensitivity analysis of tuning parameters to achieve an accurate model with a cost-effective computational run.

## Introduction

Bubble columns (BCs) refer to the contactors where a gas and liqid phase are brought into contact for the purposes of chemical reaction or separation. Indeed, a two-phase flow is created in these reactors to carry out physical and chemical phenomena such as chemical reactions. BCs, as reactors, are employed in different chemical processes like Fischer–Tropsch synthesis^1–3^, production of fine products^4^, different oxidating reactions^5,6^, alkylation reactions^7^, hazardous streams treatment^8^, gas sequestration, coal liquefaction^9^, fermentation reactions, and in cell cultures^10^, as well as the production of single-cell proteins^11^. The main benefits of bubble columns include the lack of mobile parts, easy maintenance, high heat transfer rates, and high liquid holdup being desired for the reactions of slow liquid stage^12^. The process parameters that can affect the efficiency of bubble columns, like reactors, include the distribution of gas holdup, the extent of liquid-stage back mixing, gas/liquid mass and heat transfer coefficients, gas/liquid interfacial area, bubble coalescence, re-dispersion rates, bubble-size distributions, and bubble rising velocities. The absence of full perception about the fluid dynamics makes it problematic to enhance a bubble column reactor performance through the control of operating parameters.

The necessity of establishing a logical base for interpreting the interaction of fluid dynamic variables was the main motive for dynamic studies in bubble column modeling based on computational fluid dynamics (CFD) tools during the past decades^13^. Different techniques were proposed to solve the same basic flow problem, and modeling can be done at different sophistication levels. One can prefer treating both dispersed and continuous stages as interpenetrating pseudo-continua (viz., the Euler–Euler technique, e.g.^14–18^) or the dispersed stage as discrete entities (viz., the Euler–Lagrange technique: e.g.^19–22^). A typical option is modeling such systems by Reynolds-averaged Navier–Stokes (RANS) equations and through the Eulerian-Eulerian multi-stage model^23,24^. The physical interactions between the phase stages should be modeled accurately^25^. Such interactions are governed by various interfacial forces^26,27^, and the drag force is the most significant.

Analyzing the flow field as well as temperature distribution in BCs can be conducted through experimental measurements, while measuring the pressure and velocity is highly problematic and costly in the enclosure because of low flow strength and velocity. Therefore, CFD is exploited for achieving buoyancy-induced flow and temperature fields in various shaped enclosures. However, this approach possesses significant shortcomes including accuracy and stability. Such problems happen particularly in nonlinear systems, while CFD analysis requires long computation time and high computation cost. As a result, soft programming tools (e.g. Fuzzy Inference Systems (FIS)^28–33^ and Ant Colony Optimization (ACO) algorithm) can be employed for analyzing and predicting flow, heat, and problems related to mass transfer^34–36^.

In addition, CFD computing methods can generate large datasets with several inputs and outputs (big data). This type of dataset can be a piece of interesting information for soft computing models for training. In this regard, soft computing models can easily find a connection between input and output parameters compared to discrete or global datasets that are popular in experimental observation. On the other hand, the generation of local node information enables soft computing models to describe the distribution of flow characteristics or heat and mass transfer in the reactor. Among different soft computing models, Ant Colony Optimization (ACO) can be a new option to learn non-discrete datasets as a family of swarm intelligence algorithms. This method, based on the concept of ants behavior to search an optimal path between their colony and a source of food, can train large datasets and present the distribution of datasets as a function of several input parameters. Alternatively, the ACOFIS can be presented in different technologies and applications as a problem solver or predictive tools to optimize challenging engineering processes with several operational parameters. Bubbly flow in the bubble column reactor can be an excellent candidate to present very complicated engineering processes due to flow behavior, turbulence properties, and heat characteristics which are complex to be simulated using mechanistic models.

In the current research paper, a 2-dimensional bubble column reactor with CFD was modeled and Artificial Intelligence (AI) was applied to create an artificial framework for process simulation and understanding. ACO was used in the learning stage of the AI model, and the fuzzy interface system was applied for the prediction stage based on the ant colony optimization method training. X and Y computing nodes, simulation time, liquid velocity in the Y direction, and gas hold-up are input parameters, and the temperature distribution is the output of training datasets. Different tuning parameters from ACOFIS were studied, such as cluster influence range, CIR in the training of the system, and their effects on better prediction of fluid temperature were investigated. ACOFIS is also compared with the genetic algorithm fuzzy inference system (GAFIS) and Particle swarm optimization fuzzy inference system (PSOFIS) method regarding model accuracy and ability of temperature prediction in the bubble column reactor. The training and prediction time in predicting temperature are also calculated for all models. This evaluation and analysis for different training models to build up the FIS structure in predicting reactor properties, particularly heat transfer characteristics, have been proposed for the first time. In addition to the prediction of reactor specifications with different AI models, the importance of tuning parameters in the ACOFIS is also highlighted for future investigations.

## Methodology

### CFD approach

Numerical methods and algorithms in computational fluid dynamics are employed for understanding the problems which involve fluid flows. In this study, the Euler–Euler multi-phase technique was applied to solve the average mass, flow, and energy equations and the volume fraction equation separately for each stage. The continuity equation can be defined as^37,38^:

1 $$ \frac{\partial }{\partial t}(\rho_{k} \varepsilon_{k} ) + \nabla (\rho_{k} \varepsilon_{k} u_{k} ) = 0 $$

Momentum conservation equation^37,38^:

2 $$ \frac{\partial }{\partial t}(\rho_{k} \varepsilon_{k} u_{k} ) + \nabla (\rho_{k} \varepsilon_{k} u_{k} u_{k} ) = - \nabla (\varepsilon_{k} \tau_{k} ) - \varepsilon_{k} \nabla \rho + \varepsilon_{k} \rho_{k} g + M_{I,K} $$

The stress term of gas bubbles and liquid stage can be presented by^37^:

3 $$ \tau_{k} = - \mu_{eff,k} (\nabla u_{k} ) + (\nabla u_{k} )^{T} - \frac{2}{3}I(\nabla u_{k} ) $$

In which $\mu_{eff,k}$ represents the effective viscosity. Bubble induced can be described as follows^37^:

4 $$ \mu_{eff,l} = \mu_{L} + \mu_{T,L} + \mu_{BI,L} $$

Sato et al.^39^ indicated viscosity of turbulence which was induced by the movement of multi-bubbles. Different studies applied this model for predicting the BCs^23,37,40–43^. Formulating viscosity because of the induced turbulence can be defined by the equation below^37^:

5 $$ \mu_{BI,L} = \rho_{L} C_{\mu ,BI} \varepsilon_{G} d_{B} \left| {u_{G} - u_{L} } \right| $$

With a model constant of $C_{\mu ,BI} = 0.6$ being mentioned in previous studies^37,41,44–49^.

6 $$ \mu_{eff,G} = \frac{{\rho_{G} }}{{\rho_{L} }}\mu_{eff,L} $$

The drag forces between gas and liquid are shown^37^:

7 $$ M_{D,L} = - \frac{3}{4} \in_{G} \rho_{L} \frac{{C_{D} }}{{d_{B} }} |u_{G} - u_{L} |(u_{G} - u_{L} ) $$

The Schiller–Naumann drag model can be used. In general, it is acceptable for all multi-stage measurements^50^. The drag coefficient CD is defined by Schiller and Naumann as follows^51^:

8 $$ f(x) = \left\{ {\begin{array}{ll} {24(1 + 0.15{\text{Re}}^{0.687} )/{\text{Re}} , } & {\quad {\text{Re}} \le 1000} \\ {0.44,} & {\quad {\text{Re}} > 1000} \\ \end{array} } \right. $$

The turbulent dispersion force, which was introduced by Lopez de Bertodano is defined by the following equation ^52^:

9 $$ M_{TD,L} = - M_{TD,G} = - C_{TD} \rho_{L} K\nabla \in_{L} $$

Energy conservation equation:

10 $$ \begin{gathered} \frac{{\partial (\alpha_{q} \rho_{q} H_{q} )}}{\partial t} + \nabla .\left( {\alpha_{q} \rho_{q} \overrightarrow {{v_{q} }} H_{q} } \right) = - \alpha_{q} \left( {\frac{{\partial P_{q} }}{\partial t}} \right) + \overline{{\overline{{\tau_{q} }} }} \hfill \\ :\nabla \overrightarrow {{v_{q} }} - \nabla .\overrightarrow {{q_{q} }} + S_{q} + \mathop \sum \limits_{P = 1}^{n} \left( {Q_{pq} + \dot{m}_{pq} H_{pq} - \dot{m}_{qp} H_{qp} } \right) \hfill \\ \end{gathered} $$

In which $H_{q}$ represents the enthalpy of stage q,$\overrightarrow {{q_{q} }}$ represents the heat flux, $S_{q}$ implies the heat source, $Q_{pq}$ represents the heat exchange between the pth and qth stages, $\dot{m}_{pq}$ shows the mass transfer from pth to the qth stage. The heat flux, $\overrightarrow {{q_{q} }}$ is defined as ^38^:

11 $$ \overrightarrow {{q_{q} }} = - K_{q} \partial T/\partial \vec{r} $$

In which $K_{q}$ represents the conductivity of each stage.

The energy transfer rate among the stages ($Q_{pq}$) can be assumed to be a function of the temperature difference among the stages^38^:

12 $$ Q_{pq} = h_{pq} (T_{p} - T_{q} ) $$

In which $h_{pq}$ implies the heat transfer coefficient between the pth and qth stages. The heat transfer coefficient can be associated to the pth stage Nusselt number, $Nu_{p}$, by^38^:

13 $$ h_{pq} = 6k_{q} \alpha_{p} \alpha_{q} Nu_{p} /d_{p} $$

where $K_{q}$ represents the thermal conductivity of the qth stage. In regard with the heat transfer between the liquid and the gas, this study utilized Ranz–Marshal equation^53^ which for sphere is:

14 $$ Nu_{p} = 2 + 0.6Re_{p}^{1/2} Pr_{p}^{1/3} $$

$Re_{p}$ implies the Reynolds number according to the diameter of the pth stage bubbles or particles as well as the relative velocity and $Pr_{p}$ implies the Prandtl number of the qth stage.

The turbulent eddy viscosity, the turbulent kinetic energy ($k$) and its energy dissipation rate ($\varepsilon$) are indicated in the equations below ^37^:

15 $$ \mu_{T,L} = \rho_{L} C_{\mu } \frac{{K^{2} }}{\varepsilon } $$

16 $$ \frac{\partial }{\partial t}(\rho_{L} \in_{L} K) + \nabla (\rho_{L} \in_{L} u_{L} K) = - \nabla \left( { \in_{L} \frac{{\mu_{eff,L} }}{{\sigma_{K} }}\nabla_{k} } \right) + \in_{L} (G - \rho_{L} \varepsilon ) $$

17 $$ \frac{\partial }{\partial t}(\rho_{L} \in_{L} \varepsilon ) + \nabla (\rho_{L} \in_{L} u_{L} \varepsilon ) = - \nabla \left( { \in_{L} \frac{{\mu_{eff,L} }}{{\sigma_{\varepsilon } }}\nabla_{\varepsilon } } \right) + \in_{L} \frac{\varepsilon }{K}(C_{\varepsilon 1} G - C_{\varepsilon 2} \rho_{L} \varepsilon ) $$

### Ant colony optimization (ACO) algorithm in the fuzzy inference system (FIS) training prosses

The FIS refers to a fuzzy inference system that predicts the behavior of complicated and nonlinear systems accurately^54–57^. ACO algorithm refers to AI method to solve complex problems, that can be reduced for discovering appropriate paths via the graphs being used in the fuzzy inference system training process^58–71^. In the present study X and Y computing elements, simulation time, liquid velocity distributions, and gas hold-up (gas fraction in the reactor) are input parameters, and the liquid temperature distribution is the output of training datasets. The function of the ith rule is as follows:

18 $$ {\text{w}}_{{\text{i}}} = \mu_{{{\text{Ai}}}} ({\text{X}}) \mu_{{{\text{Bi}}}} ({\text{Y}})\mu_{{{\text{Ci}}}} ({\text{Time}})\mu_{{{\text{Di}}}} ({\text{V}})\mu_{{{\text{Ei}}}} (\epsilon_{g} ) $$

where *w*_*i*_ represents the output signal of the second layer’s node and *μ*_*Ai*_, *μ*_*Bi*_, *μ*_*Ci*_, *μ*_*Di*_ and *μ*_*Ei*_ represent the input signals of the implemented MFs on the inputs, X-direction (X), Y-direction (Y), time, velocity in the Y direction (V) and a gas fraction ($\epsilon_{g}$), to the second layer's node. The third layer can be described as the following^72,73^:

19 $$ \overline{{{\text{w}}_{{\text{i}}} }} = \frac{{{\text{w}}_{{\text{i}}} }}{{\sum ({\text{w}}_{{\text{i}}} )}} $$

where $\overline{{w_{i} }}$ represents normalized firing strengths. The node function is described as follows^73^:

20 $$ \overline{{{\text{w}}_{{\text{i}}} }} {\text{f}}_{{\text{i}}} = \overline{{{\text{w}}_{{\text{i}}} }} ({\text{m}}_{{\text{i}}} {\text{X}} + {\text{n}}_{{\text{i}}} {\text{X}} + {\text{o}}_{{\text{i}}} {\text{Y}} + {\text{p}}_{{\text{i}}} {\text{Y}} + {\text{q}}_{{\text{i}}} {\text{Y}} + {\text{r}}_{{\text{i}}} ) $$

In which *m*_*i*_, *n*_*i*_, *o*_*i*_, *p*_*i*_, *q*_*i,*_ and *r*_*i*_ represent the if–then rules’ parameters named as consequent parameters. The training datasets can be defined in the structure of a fuzzy system by the distribution of membership functions. In this regard, several membership functions can be defined in each input parameter to fully translate numbers and train datasets into the function shape with different function distribution across input parameters. All the input signals from the fourth layer were aggregated to achieve the model output representing the result of estimation.

The FIS structure is designed to implement the final decision part of the model, which represent the conceptual understanding of human^74–76^. This structure has been previously described by Takagi, Sugeno, and Kang. The main training part of FIS can also be defined with different algorithms for a better understanding of physical processes. These learning methods contain several model parameters or tuning options to improve the model’s accuracy, prediction capability and even reduce the computational learning time. The difference between the FIS structure is based on the weighted average of the rule output parameters rather than the max operator mechanism. Moreover, the rule output with defuzzification analysis can generate a model with inexpensive computational requirements. This cost-effective model is called the Sugeno model^77^.

### Model comparison

For better evaluation of the ACOFIS model, this prediction algorithm is compared with GAFIS and PSOFIS models in similar modeling conditions (see Table 1). Five inputs are selected in all models, while 75% of all datasets have participated in the training models. All models are trained with 70 number of epoch or iteration number. The Sugeno type is the FIS structure for all models. Additionally, subtractive clustering is used as a clustering type in the model with a cluster influence range of 0.5 for all models^36^. The reject ratio as a subtractive clustering parameter is similar for GAFIS, PSOFIS, and ACOFIS models. In the next step of the selection of model parameters, the pheromone effect and number of ants for ACO are 0.2 and 20, respectively. For the GA method, other parameters are selected such as swarm size = 20, crossover percentage = 0.7, and mutation percentage = 0.5. Finally, for the PSO model population size, the inertia, weight damping ratio, personal learning coefficient, and global learning coefficient are 20, 0.99, 1, and 2, respectively.

**Table 1:** Model parameters in the generation of FIS and clustering classification for ACO, GAFIS, PSOFIS algorithms.

Methods	ACOFIS	GAFIS	PSOFIS
Number of inputs	5	5	5
Maximum of iteration	70	70	70
Percentage of data in training process	75	75	75
Type of FIS	Sugeno	Sugeno	Sugeno
Clustering type	Subtractive Clustering	Subtractive Clustering	Subtractive Clustering
Cluster influence range as subtractive clustering parameter	0.5	0.5	0.5
Accept ratio as subtractive clustering parameter	0.5	0.5	0.5
Reject ratio as subtractive clustering parameter	0.15	0.15	0.15
Pheromone effect as ACO parameter	0.2	–	–
Number of ants as ACO parameter	20	–	–
Swarm size as GA parameter	–	20	–
Crossover percentage as GA parameter	–	0.7	–
Mutation percentage as GA parameter	–	0.5	–
Population size as PSO parameter	–	–	20
Inertia weight damping ratio as PSO parameter	–	–	0.99
Personal learning coefficient as PSO parameter	–	–	1
Global learning coefficient as PSO parameter	–	–	2

## Results and discussion

**Figure 1:**
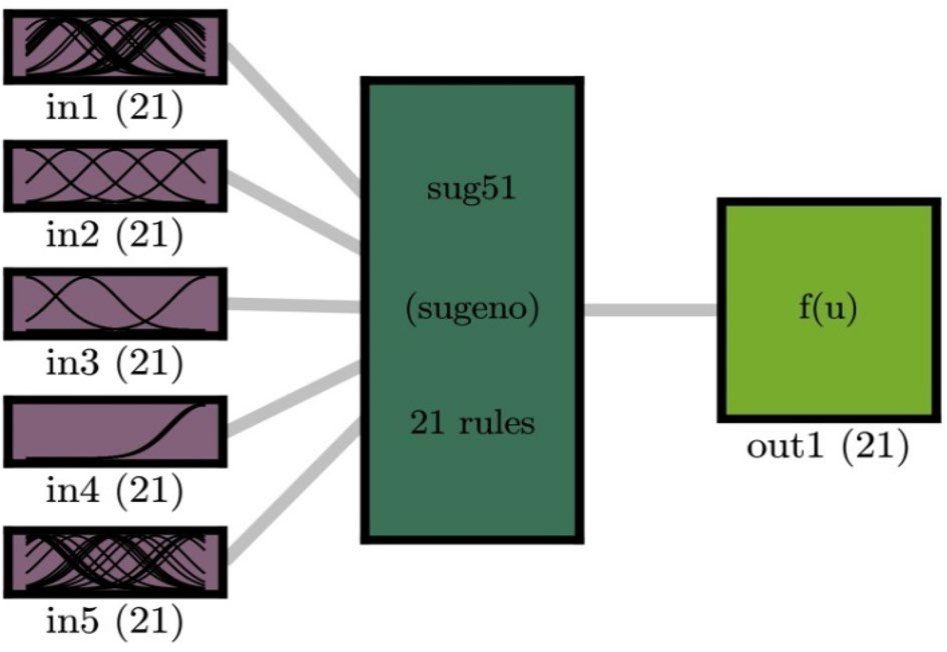
FIS system based on ACO algorithm in the learning process, using subtractive clustering in the best of ACOFIS intelligence when CIR is 0.5, number of ants is 20, and the pheromone effect is 0.2.

We used 75% of the data for training processes. The remained 25% of the data plus the 75% in the training were studied in the evaluation or testing step of simulations. For clustering the data, we used subtractive clustering with the *gaussmf* membership function. As mentioned earlier, we used five inputs for the training process. In the domain of ant colony optimization method, we used 20 number of ants numbers so that we could reach the best intelligence in the system, and for the general evaluation of the system, we used the regression system. As shown in Fig. 1, we have five inputs and one output, which is the fluid temperature in the training process. After the training process, the trained datasets are translated into the fuzzy structure based on membership specifications. The membership functions can translate all learning processes in the fuzzy structure and train the main FIS structure. The ant colony optimization method is selected as the main learning algorithm. For the selection of membership function, in general, in the subtractive clustering method, when the CIR number is defined in the method number of the membership function is dictated for each input parameter. For example, as Fig. 1 shows, by selecting CIR number 0.5, several membership functions (21 membership functions) are distributed in each input parameter. However, decreasing the number of CIR in the method number of membership functions rises in each input parameter, representing a direct correlation between the number of CIR and membership functions. In this regard, to select the correct number of CIR, the sensitivity analysis is implemented in this study to evaluate the level of model accuracy and learning computational time.

**Figure 2:**
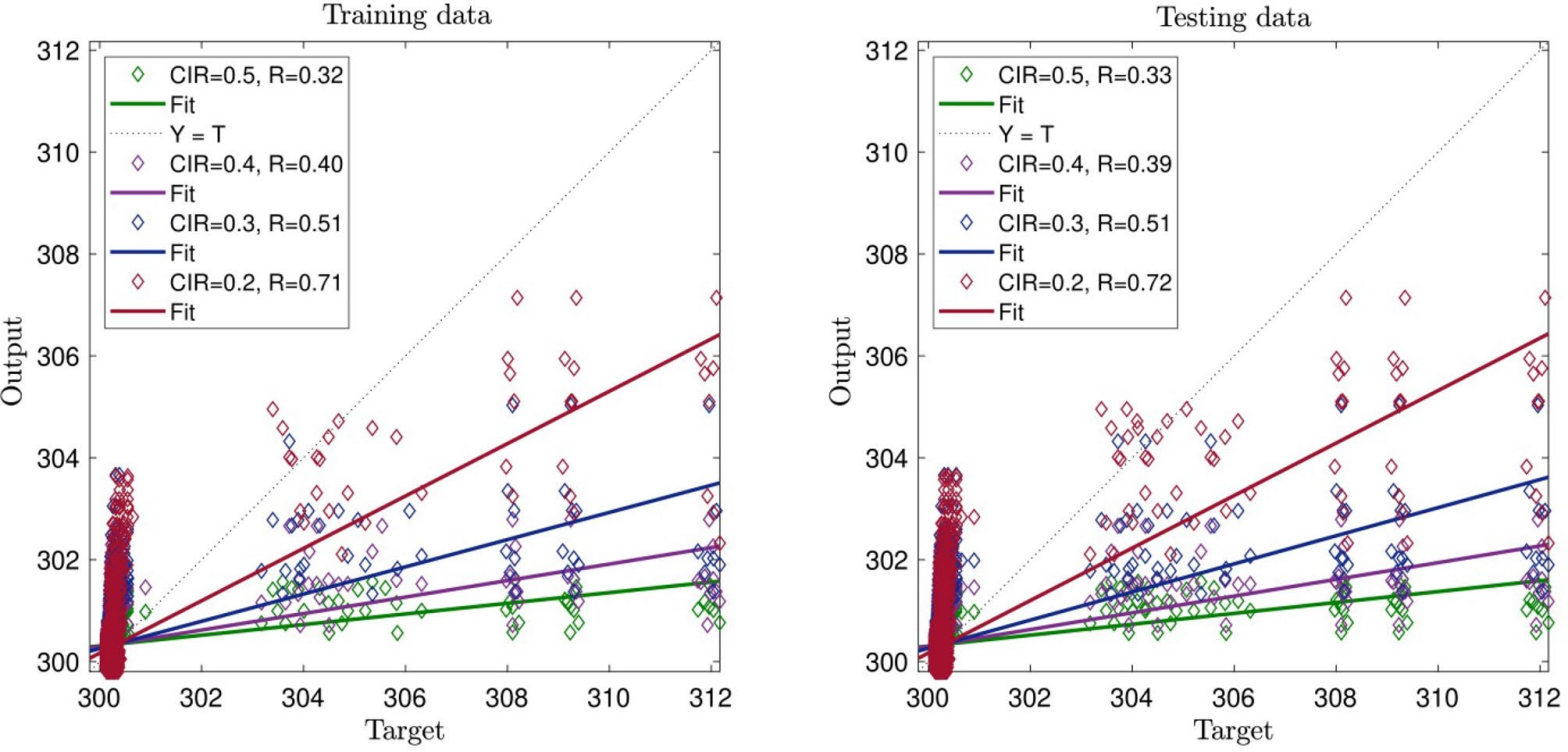
CIR (clustering parameter) changes in the ACOFIS learning processes when the pheromone effect is 0.2 and number of inputs is 2.

For analysis, we evaluate both testing and training data. For example, as shown in Fig. 2, we put 3000 points in the training process, and used 20 numbers of ants, as shown *R* reaches 0.71. The data used in the training process was also used in the testing process, and our evaluation *R* reaches 0.72, which is very close to the evaluation level in the training process.

**Figure 3:**
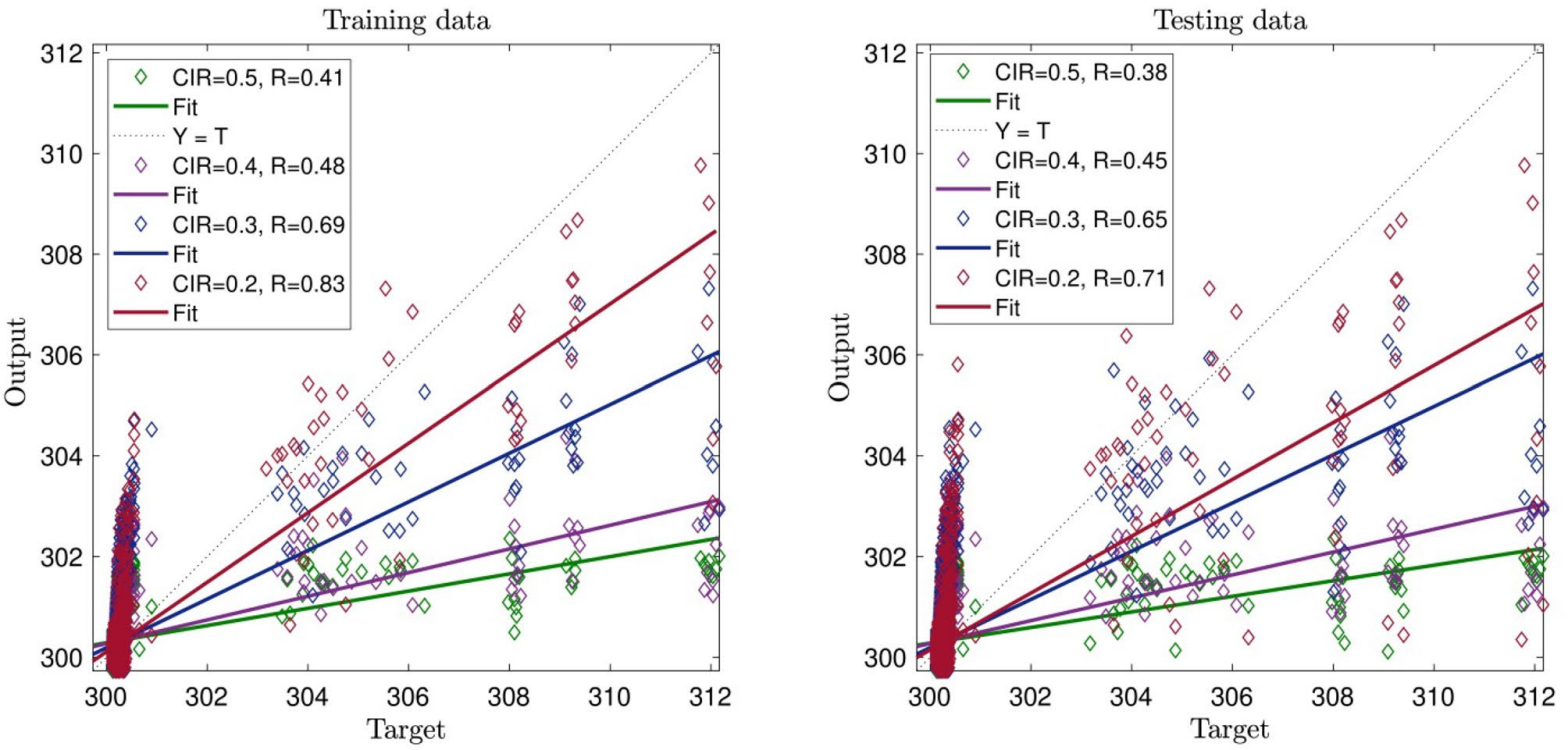
CIR (clustering parameter) changes in the ACOFIS learning processes when the pheromone effect is 0.2 and number of inputs is 3.

Despite the previous data, we increase CIR, and therefore, *R* decreases significantly, and the system does not show an intelligent signal (prediction capability) in Fig. 2. By increasing CIR, as shown in Fig. 2, the intelligence of the system is decreasing. With CIR number 0.4, the intelligence of the system reachers *R* = 0.4 , and the testing and training processes are very close to each other. Finally, with CIR number 0.5, the intelligence of the system reaches its lowest amount that is *R* = 0.3. Figure 3 shows the three inputs in the training process with the CIR number 0.2. In this regard, the system reaches an average intelligence which is 0.83.

**Figure 4:**
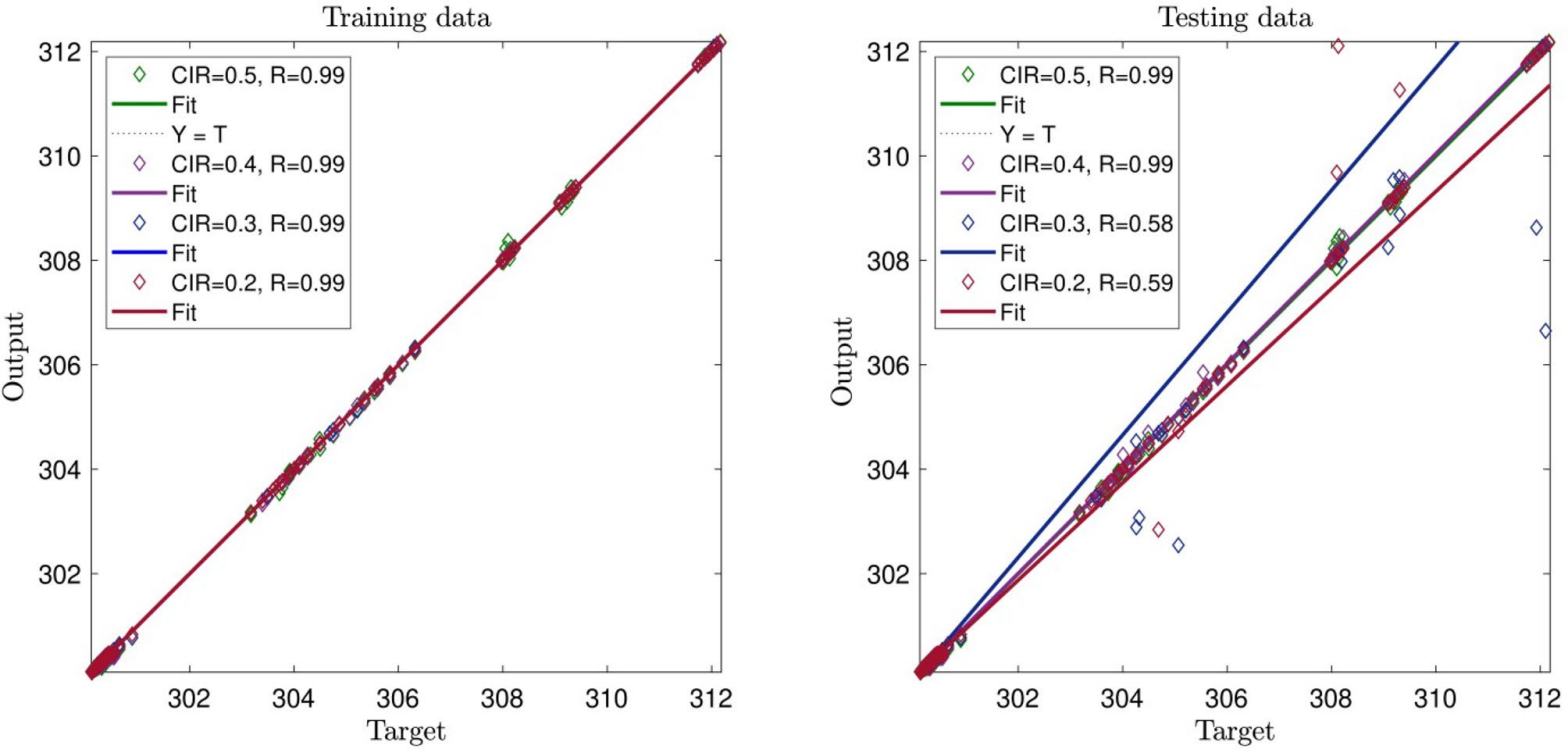
CIR (clustering parameter) changes in the ACOFIS learning processes when the pheromone effect is 0.2 and number of inputs is 4.

In this domain, by increasing the system’s CIR to 0.5, the system’s intelligence starts decreasing; however, comparing it to the previous Figure, the amount of decrease is lower. As shown in Fig. 4, the system’s intelligence increases significantly in both training and testing processes. In this part, we studied four inputs for the AI system, and from the beginning with CIR number 0.2, the intelligence of the system reaches 0.99 in both training and testing. The signal relating to the intelligence of the system reveals that by increasing the number of inputs in the system, we can significantly increase its intelligence. As such, the number of inputs plays a key role in the intelligence of the system, so increasing the number of inputs can increase the intelligence of the system. In continuation of explaining Fig. 4, despite increasing the CIR number to 0.5, the intelligence of the system does not tend to decrease, emphasizing the point that the system reaches its full amount of intelligence, and the parameters that previously affect the intelligence of the system negatively seems to become neutral.

**Figure 5:**
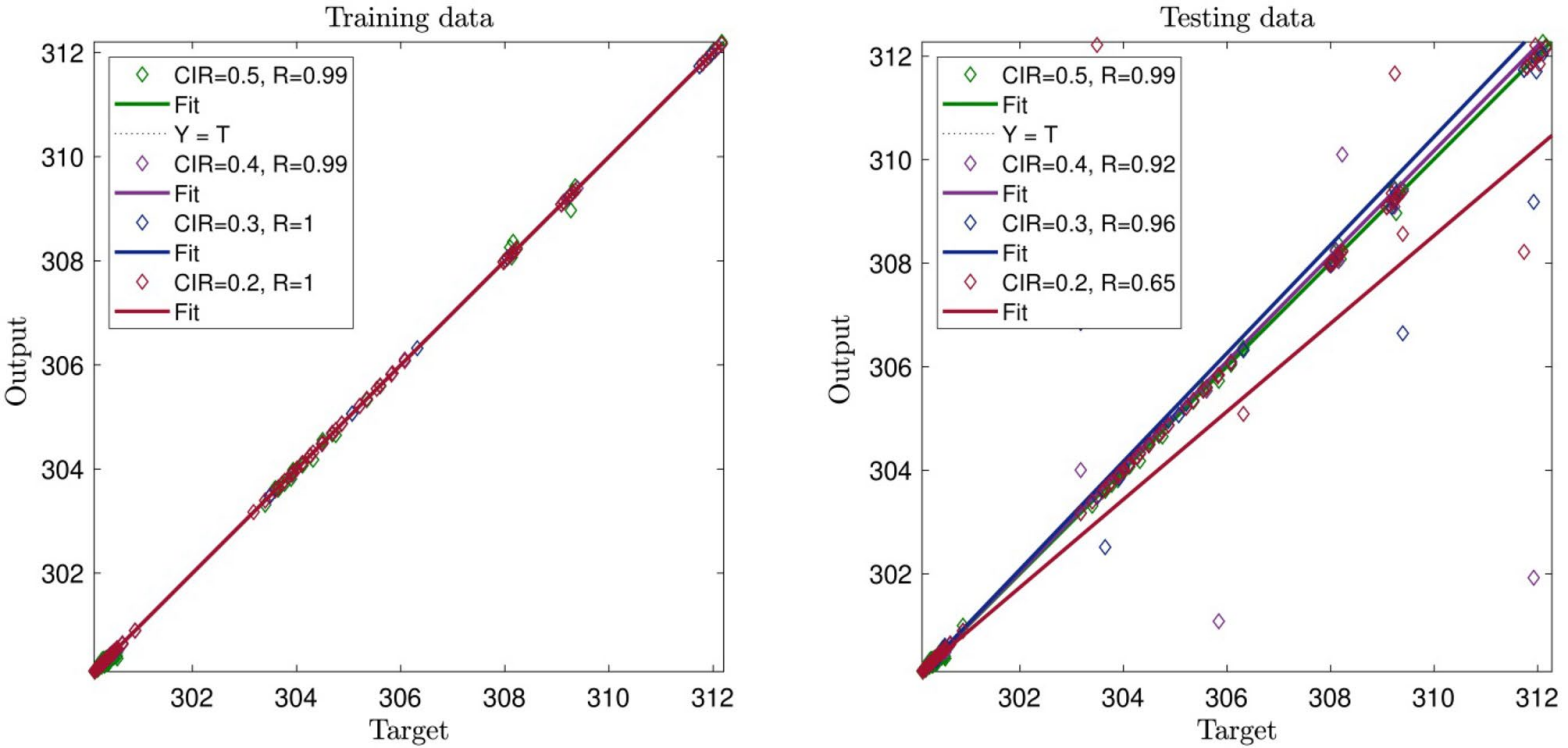
CIR (clustering parameter) changes in the ACOFIS learning processes when the pheromone effect is 0.2 and number of inputs is 5.

Figure 5 shows interesting results. By enhancing the number of inputs, the network becomes greatly intelligent, and the *R* reaches unity. By increasing the CIR of the system, the number stays the same. The results also show that when the number of input is smaller than four, CIR number should be small. In general small CIR, number requires expensive computational runs and calculations. However, when the input number is four or five, we can observe different behavior in the model. In this case, the model can accurately predict datasets with a high number of CIR (0.5) with lower computational time.

**Figure 6:**
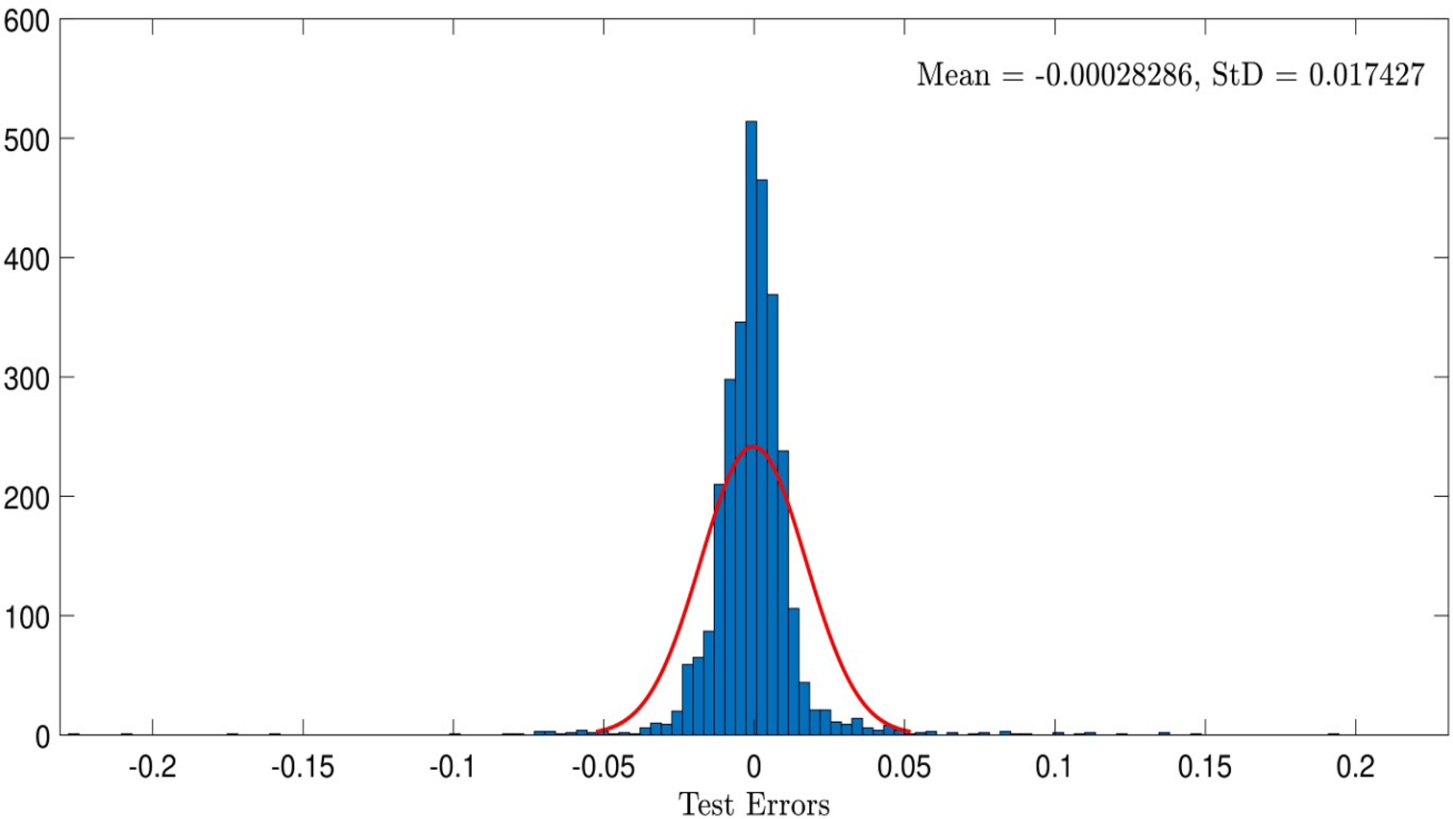
Testing errors histogram in the best of intelligence when CIR is 0.5, number of ants is 20, and the pheromone effect is 0.2.

**Figure 7:**
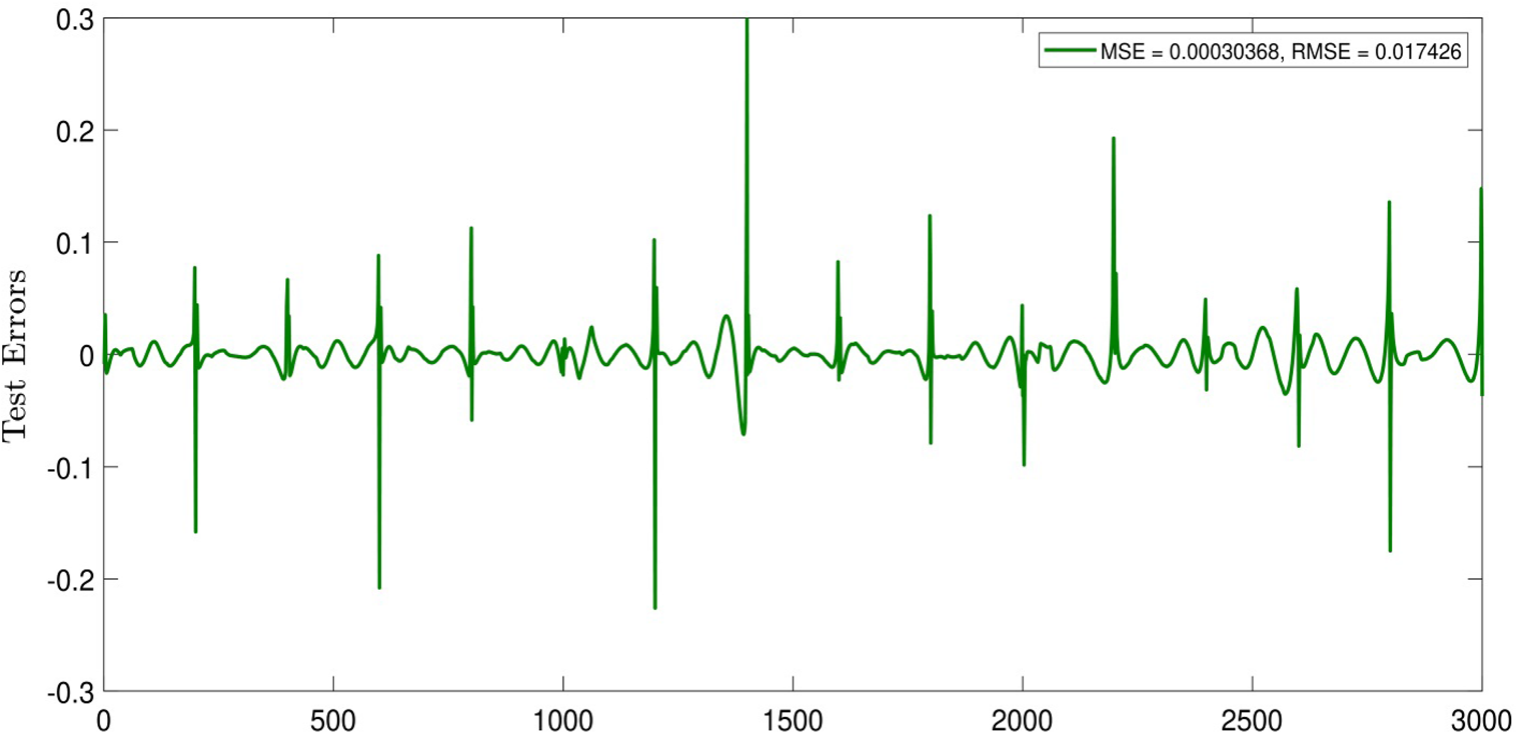
Test_ErrorsnewACO, nIP = 5, CIR = 0.5, ndata = 3000, Subtractive, MaxEpoch = 70, P = 75, nant = 20, q = 0.2, CIR = 0.2.

For better understanding the intelligence of the system, we study an error distribution diagram. As shown in Fig. 6, the error relating to zero has the highest amount of aggregation, and when we have such a diagram in the middle of a graph, by aggregation of the points in zero, it shows an intelligent system. This visualization was done for CIR number 0.5 and 5 inputs in the system. Also, we plotted a general error for each of the data sets, so that we could observe the amount of error in the system. We face an oscillation of errors, but it does not decrease the swarm intelligence of the system (see Fig. 7).

**Figure 8:**
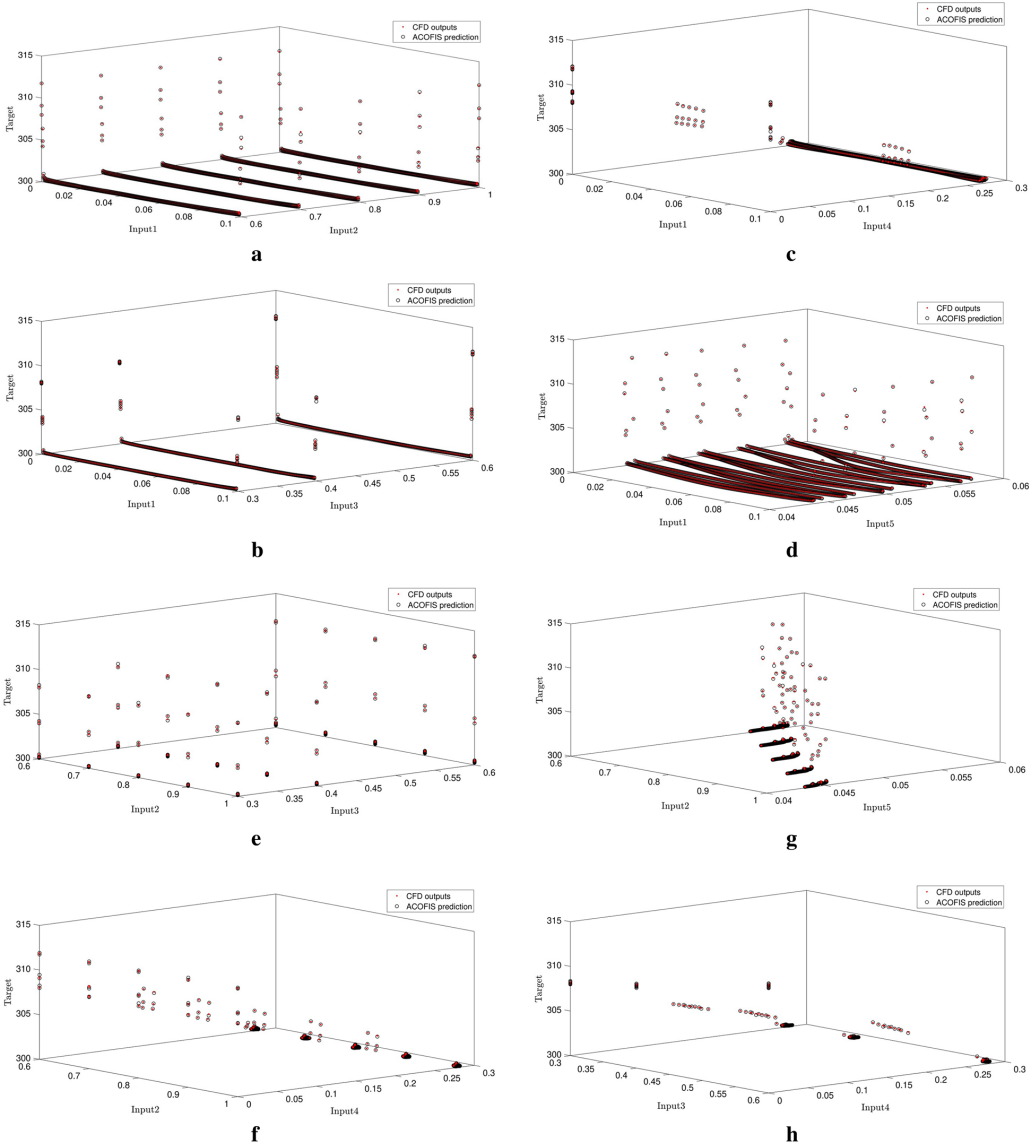

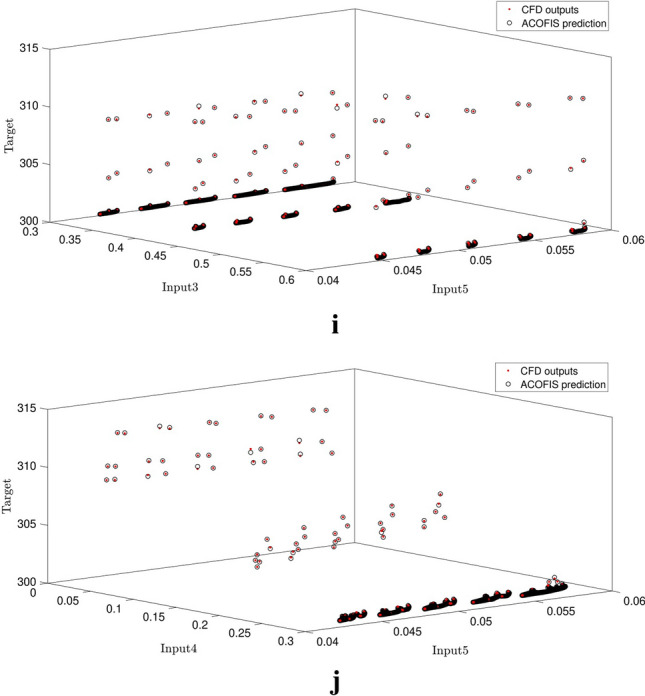
Liquid temperature prediction by high level of ACOFIS intelligence when pheromone effect is 0.2 and CIR is 0.5 based on each two inputs.

Figure 8a–j show the inputs comparing to each other. As shown in the Figures, the CFD results are completely in agreement with the numerical results of swarm intelligence and fuzzy logic system. Results from the ACOFIS system can accurately predict the CFD results which are shown in Fig. 8.

**Figure 9:**
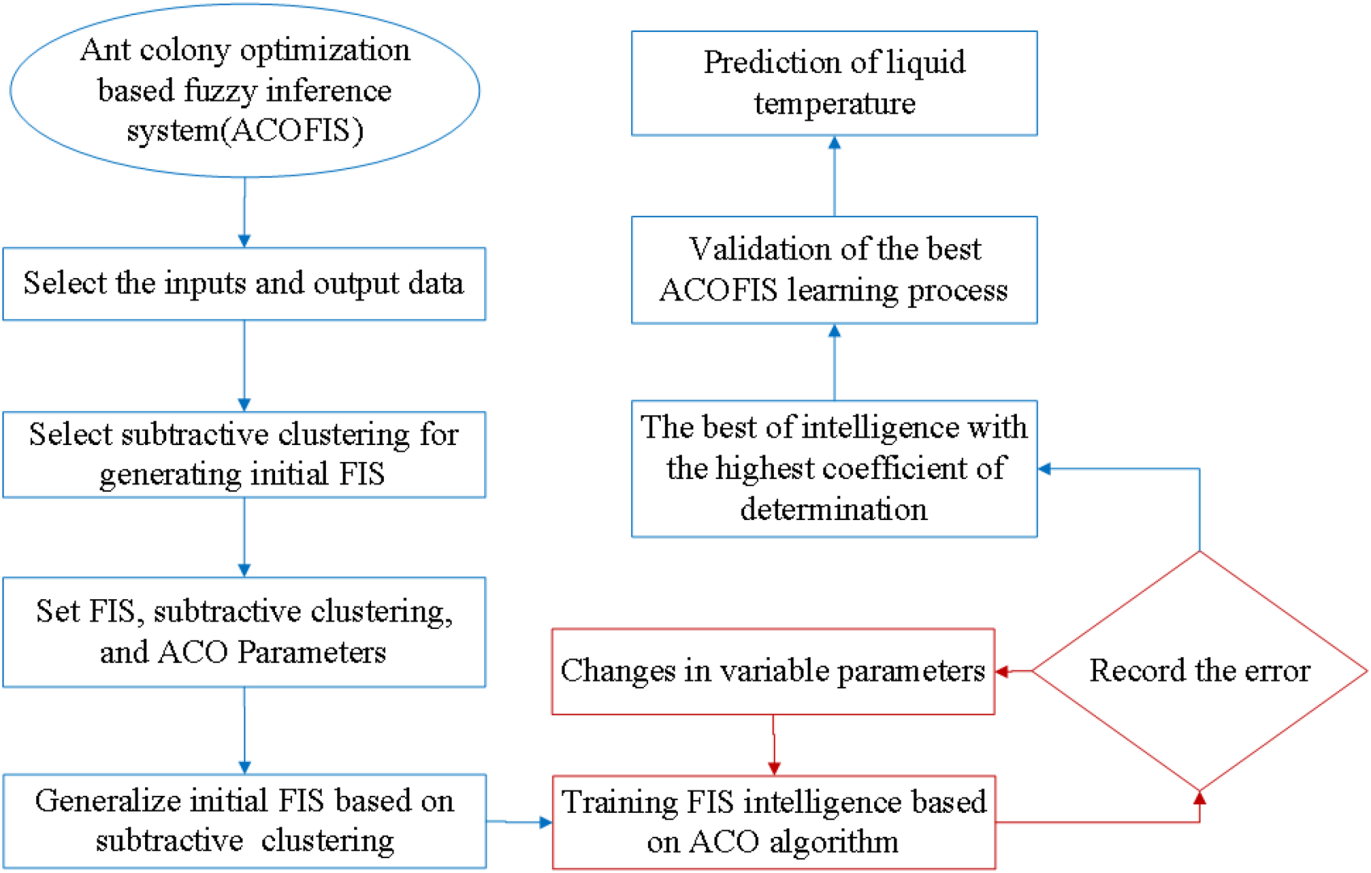
Flowchart for ACOFIS method, including ACO training part and generation of the FIS structure.

To understand the main algorithm and all assessment criteria and check the model accuracy and prediction capability the designed flow chart is illustrated in Fig. 9. In the initial steps of running ACOFIS, the input parameters and output parameters are selected in the model. The subtractive clustering is defined to build up the main FIS structure. Then, all parameters of subtractive clustering and ACO are described in the algorithm. In the next step, the FIS structure is generated based on subtractive clustering. To design (train) the FIS structure, the ant colony optimization method is used. However, to increase the ability of method in learning datasets, errors are recorded in the method, and if the error is not sufficient to achieve the high level of accuracy or prediction capability, model parameters are changed for another training process. In the case of having a small error in the model, the coefficient of determination is evaluated for the model, and the trained model is validated based on “*non-trained*” datasets. In the final stage of the method, the method’s prediction section is evaluated to show the liquid temperature distribution in the domain of calculation.

**Figure 10:**
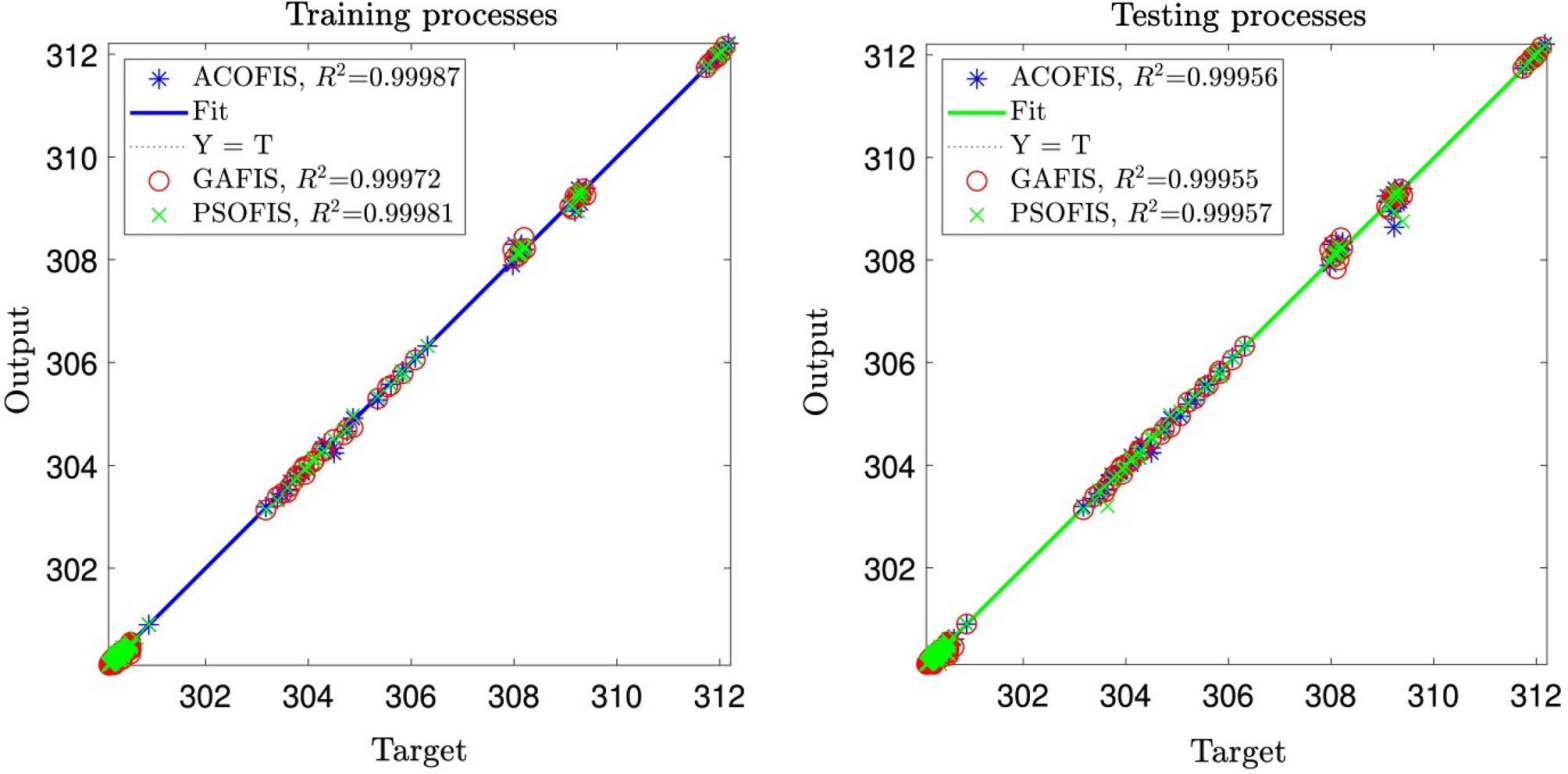
Comparison of coefficient of determination for training and testing processes for different methods.

**Figure 11:**
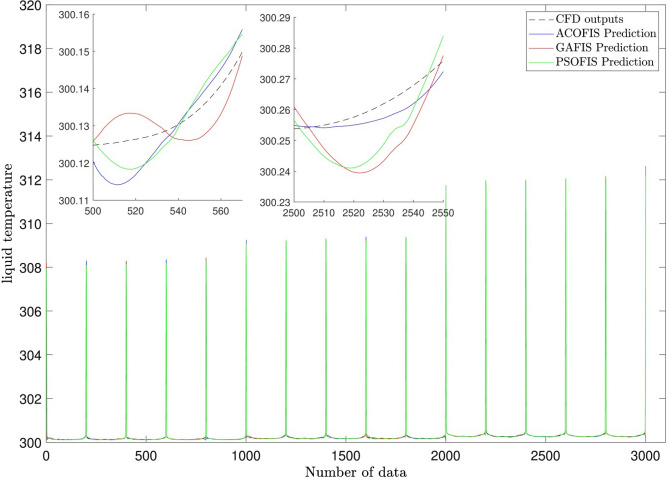
Prediction of liquid temperature in the bubble column reactor by ACOFIS, GAFIS, PSOFIS methods.

For better evaluation of the current training model (ACO) this model is compared with other training algorithms, such as GA and PSO with respect to model accuracy, coefficient of determination (R^2^). Figure 10 shows the comparison between models for training and testing processes for different training models generating FIS structure. *R*^2^ for all models are very high $R^{2} > 0.999$ for both training and testing. However, for better evaluation of prediction capability, temperature distribution as a function of the number of data can be considered. Figure 11 shows the prediction capability for ACOFIS, GAFIS, and PSOFIS models. The liquid temperature in the reactor is predicted with different models. The results show that all models are very accurate in predicting the liquid temperature of the reactor. However, there is a small discrepancy in predicting local points for all machine learning models. This difference does not have an impact on the overall prediction of temperature distribution in the reactor. To thoroughly analyze the difference between machine learning methods, various assessment methods are considered such as MSE, RMSE, mean error, StD, *R* and *R*^2^ for the training and testing processes. In addition to that, training time and prediction time are computed for all methods and compared together (see Table 2). The results show that all methods are similar with regards to model accuracy and evaluation criteria. For example, *R* and *R*^2^ for ACOFIS, PSOFIS, and GAFIS in the training and testing processes are more than 0.999. A similar finding is achieved for other error evaluation parameters, such as MSE and RSME, and STD. Table 2 also shows that the PSOFIS model can train datasets in half a time compared by ACOFIS. However, the prediction time for all models is less than one second. These results show that all ACOFIS, PSOFIS, and GAFIS models can be a good replacement prediction model instead of CFD computing models in estimating the temperature in the bubble column reactor. Table 1 shows all model parameters for different machine learning models, ACOFIS, GAFIS, and PSOFIS.

**Table 2:** Different error approaches (training and testing processes), learning and prediction times for ACO, GAFIS, PSOFIS algorithms.

Methods	ACOFIS	GAFIS	PSOFIS
Training MSE error	0.000323559	0.000346098	0.000249941
Training RMSE error	0.017987748	0.018603713	0.015809534
Training mean error	− 4.92416E−13	− 4.40021E−12	− 1.24685E−11
Training standard deviation (StD)	0.017991747	0.018607849	0.015813048
Training correlation coefficient (R)	0.999867292	0.999857902	0.999903986
Training coefficient of determination (R^2^)	0.999734602	0.999715825	0.999807981
Testing MSE error	0.000498202	0.000509233	0.00048397
Testing RMSE error	0.022320443	0.02256619	0.021999327
Testing mean error	0.000165595	6.04664E-05	0.00059781
Testing standard deviation (StD)	0.02232355	0.022569871	0.021994869
Testing correlation coefficient (R)	0.999778489	0.999773376	0.99978659
Testing coefficient of determination (R^2^)	0.999557028	0.999546804	0.999573225
Learning time (s)	455.4656429	268.6194803	244.3158696
Prediction time (s)	0.7167073	0.7049477	0.913323

## Conclusion

Generally, the swarm intelligence and the fuzzy logic system can be a prediction model for physical and chemical processes. In the current study, we make an AI model from a multi-phase flow in the 2-dimensional bubble column reactor data generated by CFD. In the BCR, we used the heating sources and created them in the CFD model. The data from CFD used in the ACOFIS model for training and prediction processes. Moreover, in the machine learning area, we made a FIS structure based on the training of the ACO model. As far as ant colony optimization is a local point optimization system and used to optimize discrete problems, we design it to optimize a group of data in a continuous domain. The swarm intelligence or ant colony can provide a good capability for the fuzzy interface system in terms of training processes. We used five inputs and one output in the training process to see this capability in the system. The output was the temperature of the fluid in the reactor. In the domain of sensitivity of the study, we take advantage of the effects of the cluster range in the model. After the training and prediction, we came to this conclusion that by increasing the number of inputs in the system, the system reaches a high swarm intelligence. Like the ants that they find their path to their food and optimize their path, the system can optimize the prediction of the fluid flow very fast. This is a transformation concept from ant behavior to bubbly flow behavior in the reactor. Therefore, increasing the number of inputs in the system can increase the swarm intelligence of the ants. For designing a machine learning system, we need to add the inputs as much as we can so that a system can reach a high intelligence. Sometimes, the inputs are not completely related to the system, but the AI system can find a meaningful relationship between the inputs and the outputs. The sensitivity analysis (tuning model) parameters can impact the accuracy and the overall prediction capability. Alternatively, the tuning model can make the model cost-effective with regards to learning computational time. The result shows that the small number of CIR (such as 0.2) can improve the model’s accuracy up to three input parameters. However, as the number of inputs increases, such as four or five, the small CIR number (0.2) makes the inaccurate model with an expensive computational run (high learning time). In this case, CIR number 0.5 can generate a cost-effective model with a high level of accuracy. For better evaluation of the ACOFIS model, this machine learning algorithm is compared with GAFIS and PSOFIS models in terms of model accuracy, prediction capability, training time, and prediction time. It was found that the ACOFIS model is very accurate, along with GAFIS and PSOFIS models. Additionally, all machine learning models can predict the processes in less than one second after the reactor’s temperature distribution training. However, the PSOFIS model can train the datasets in a shorter time than ACOFIS and GAFIS models. As far as this approach is a meshless method, except for the 100% data which were studied, we can create a meshless domain except for the inputs so that the prediction process can be studied in this domain. For example, x and y-direction positions make a square or a rectangle in a 2-dimensional domain, and the meshes could show thousands of points in themselves. We can create millions of data via AI, but the point worth mentioning here is that the data could be created in a particular domain, a square or a rectangle. On the other hand, millions of data that are created in the domain could be different from previous points.
